# Thermodynamic mechanism evaluate the feasibility of oil shale pyrolysis by topochemical heat

**DOI:** 10.1038/s41598-021-84757-x

**Published:** 2021-03-08

**Authors:** Shuai Zhao, Xiaoshu Lü, Youhong Sun, Jiandong Huang

**Affiliations:** 1 School of Mines, China University of Mining and Technology, XuZhou, 221116 China; 2 Construction Engineering College of JiLin University, ChangChun, 130000 China

**Keywords:** Chemical engineering, Fossil fuels, Petrol

## Abstract

Topochemical heat in-situ pyrolysis of oil shale is achieved by injecting high temperature nitrogen to promote oil shale pyrolysis and release heat, and then injecting air to trigger oil shale combustion in the early stage of oil shale pyrolysis, and then by injecting normal temperature air continuously to promote local oxidation of oil shale in the later stage. In order to verify the oil and gas recovery by topochemical heat method, Jilin University has chosen Fuyu City, Jilin Province, to carry out pilot project of oil shale in-situ pyrolysis by topochemical heat method. Besides, in order to infer the spontaneity, feasibility and difficulty of continuous pyrolysis of oil shale based on topochemical heat, this paper, the mechanism of solid-state pyrolysis and the thermodynamic analysis of transition state of oil shale in Fuyu area are discussed. Because the second stage of oil shale pyrolysis is the main stage of oil production. Therefore, the characteristics of Gibbs free energy, free enthalpy and free entropy of transition state in the main oil production stage of oil shale pyrolysis are obtained by calculation. The results show that in situ pyrolysis of oil shale topochemical heat can be carried out spontaneously and continuously, and the release characteristics of volatiles during pyrolysis of oil shale are described.

## Introduction

Oil shale is an immature hydrocarbon-generating medium. It can produce oil and gas only when the temperature reaches the pyrolysis temperature of kerogen^1^. The traditional pyrolysis mode of oil shale is mainly surface retorting process. Because surface retorting needs to establish surface factories, the one-time economic input cost is high. In addition, surface retorting process is easy to cause environmental pollution, slag accumulation and other problems. At present, the main production methods of oil shale are changing from surface distillation to in-situ pyrolysis^2,27^. Sun Youhong Research Group of Jilin University, in cooperation with the Asian Science and Technology Company of Israel, proposed TS-A in-situ pyrolysis process of oil shale based on the original topochemical pyrolysis process (TS), and prepared to carry out in-situ production test in Fuyu area of Jilin Province^3^.

**Figure 1:**
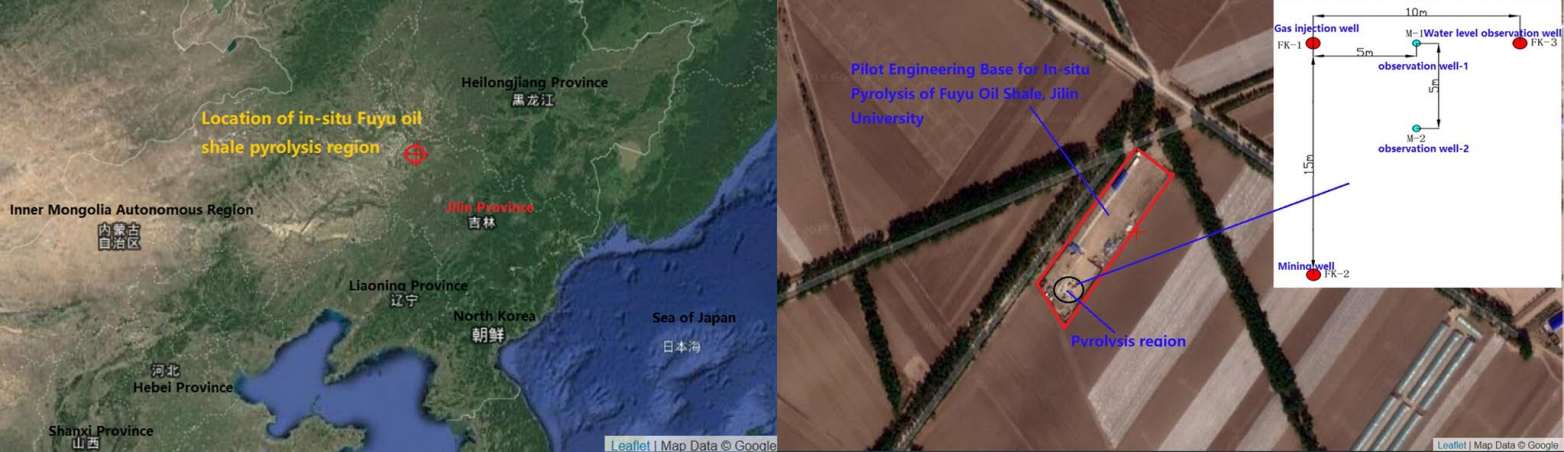
Location of oil shale in-situ pyrolysis region and layout of wells.

Topo-chemical heat in-situ pyrolysis of oil shale is a low energy consumption in-situ retorting technology. As shown in Fig. 1, it is neither simple physical heating nor completely underground combustion, but a process of chemical heat intensification triggered by topo-chemical reactions^4^. The basic principle of oil shale in-situ pyrolysis by topochemical method is to form a local columnar micro-reaction unit by injecting hot air around the well, and the range of the unit will gradually expand with the continuous decomposition of kerogen. Local oxidation occurs in oil shale when a small amount of air is involved, which can provide a lot of heat for further pyrolysis of oil shale. This not only saves energy (e.g. electricity) and reduces costs, but also makes oil shale decompose more thoroughly. However, the feasibility of Topo-chemical heat pyrolysis oil shale and the degree of difficulty of spontaneous reaction need to be discussed in depth from the mechanism.

The pyrolysis process of oil shale is a multiphase and multistage coupled chemical reaction. Zhang et al. research showed that the range of variability in the principal activation energy is from about 200 to 242 kJ mol^−1^, with most samples being in the middle half of that range, while the range of frequency factors most likely in the 10^12^–10^16^ s^−1^range, with most values within the middle half of that range^5^. E C Moine et al. studied raw oil shale in the Moroccan Rif region, and showed that the thermal decomposition mechanism of Moroccan Rif region's primary oil shale can be described by n-order (n = 1.550), and the activation energy was calculated by Kissinger–Akahira–Sunose(KAS)method. In addition, the transition state free Gibbs energy ΔG^≠^, free enthalpy ΔH^≠^ and free entropy ΔS^≠^ indicated that the pyrolysis of oil shale is non-spontaneous at low temperature^6^. Kuang et al. studied the carbon oxidation and pyrolysis process of Green River oil shale by non-isothermal thermogravimetric analysis. The extensive applicability of solid-state pyrolysis mechanism was verified by various kinetic calculation models^7^. Wei Wang et al. studied North Korean oil shale under non-isothermal conditions by thermogravimetric analysis. The results showed that the parameters of kinetic reaction order 0.5, 1 and 1.5 are suitable for describing the pyrolysis process of North Korean oil shale^8^. Sun and Bai et al. simulated the process of oil shale pyrolysis by topochemical heat under atmospheric pressure. The results show that topochemical heat can increase the original temperature of oil shale to 250–300 °C. Although the apparent temperature is analyzed in terms of heat and mass transfer, it does not explain the difficulty of reaction from kinetic and thermodynamic mechanism, and there is no in-depth explanation of the reaction mechanism. In-situ pyrolysis of oil shale is carried out in complex underground environment and working conditions. Only by analyzing the mechanism of the method, can it provide a reference for the actual field test^20,21^.

In this paper, oil shale samples came from Fuyu, Jilin Province, where a pilot project of oil shale in-situ pyrolysis by topochemical heat was prepared. The air atmosphere was used as the pyrolysis atmosphere of oil shale during the experiment. The feasibility of topochemical heat to pyrolysis oil shale method in this area was inferred by means of kinetic and thermodynamic calculation. The activation energies of oil shale pyrolysis were calculated by Kissinger–Akahira–Sunose (KAS) method, Flynn–Wall–Ozawa (FWO) method and Friedman method, respectively. On this basis, the thermal decomposition mechanism was deduced by Malek method. The reaction order n and pre-exponential factor A were calculated. Based on the transition state theory, the thermodynamic decomposition properties of oil shale in the process of low oxygen viscous flow transition are calculated by Eyring equation. In addition, free Gibbs energy ΔG^≠^, free enthalpy ΔH^≠^ and free entropy ΔS^≠^ of the transition state in the second stage of pyrolysis are calculated. It can be used to infer the spontaneous of oil shale pyrolysis at high temperature. This provides a reference for oil shale in-situ pyrolysis by topochemical heat.

## Material and principle

### Materials

Jilin University selected Fuyu City of Jilin Province as the experimental base for pilot project of oil shale in-situ pyrolysis, as shown in Fig. 1 and drilled three wells. FK-1 is an injection well for high pressure air, FK-2 is a production well, M-1and M-2 are two real-time monitoring wells of formation temperature and pressure changes, and FK-3 is a water level monitoring well for real-time monitoring of water level change in gas seepage control Area.

Among them, two wells were drilled without coring, and only FK-3 well were drilled with coring. After analyzing the core of FK-3 well, it is found that the burial depth of Fuyu oil shale is 477–486 m. Under this burial depth, two core samples of FK-3 Well are selected for industrial analysis, element analysis and Fisher analysis, which are located on the roof and floor of oil shale reservoir respectively. The samples were ground before the test. In order to avoid the influence of different particle sizes on the test results, the grinded oil shale samples were sieved into uniform particle sizes. Dry in a constant temperature drying oven at 60 °C to a constant weight. The results are shown in Table 1.

**Table 1:** Analysis of oil shale in Fuyu.

Attribute	Proximate analysis/wt%				Fisher analysis/wt%				Element analysis/wt%			
Region	Moisture	Ash	Volatiles	Fixed carbon	Shale oil	Water	Residue	Gas	H	C	N	S
Sample1	3.75	69.63	21.37	5.25	4.16	8.85	83.32	3.67	7.27	4.96	0.34	1.09
Sample2	3.68	70.07	20.99	5.26	3.97	8.36	84.08	3.59	7.02	4.86	0.32	1.12

### Thermogravimetry test method

**Figure 2:**
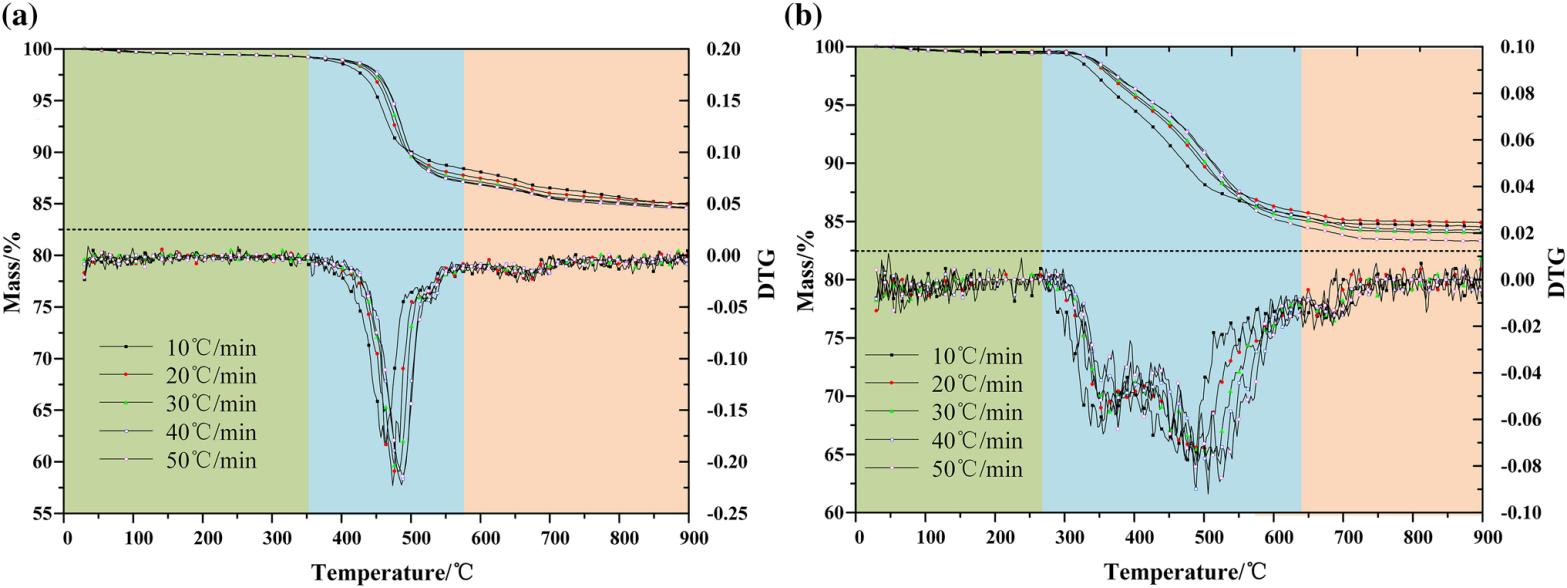
TG-DTG curve of Fuyu oil shale (**a**) nitrogen, (**b**) air.

The weight of samples was controlled at 9.0(± 0.2 mg), in which the initial temperature of TG curve was 25 °C, the heating rate was 10, 20, 30, 40, 50  °C/min, and the reaction termination temperature was 900 °C. The purge gas is nitrogen and air with flow rate of 60 mL/min and the protective gas is high purity nitrogen with flow rate of 25 mL/min. In order to reproduce and reproduce the experiment, each group of experiments was repeated at least twice. The TG-DTG curve of Fuyu oil shale pyrolysis in nitrogen and air was shown in Fig. 2.

According to thermogravimetric curves, oil shale pyrolysis can be divided into three stages, but the main oil generation stage is the second stage of oil shale pyrolysis. In the second reaction stage. Temperature ranges from 300 to 620 °C, and the weight loss rate of oil shale is about 15% to 17% in this stage. With the increase of heating rate, the pyrolysis process of oil shale becomes more intense. According to proximate analysis data, the first stage of oil shale pyrolysis was the process of free water loss, and the weight loss rate of this process is about 1.2–1.6%. The third stage was mainly the decomposition process of clay minerals under high temperature. The weight loss rate of this process is slow, about 2–2.5%.

**Figure 3:**
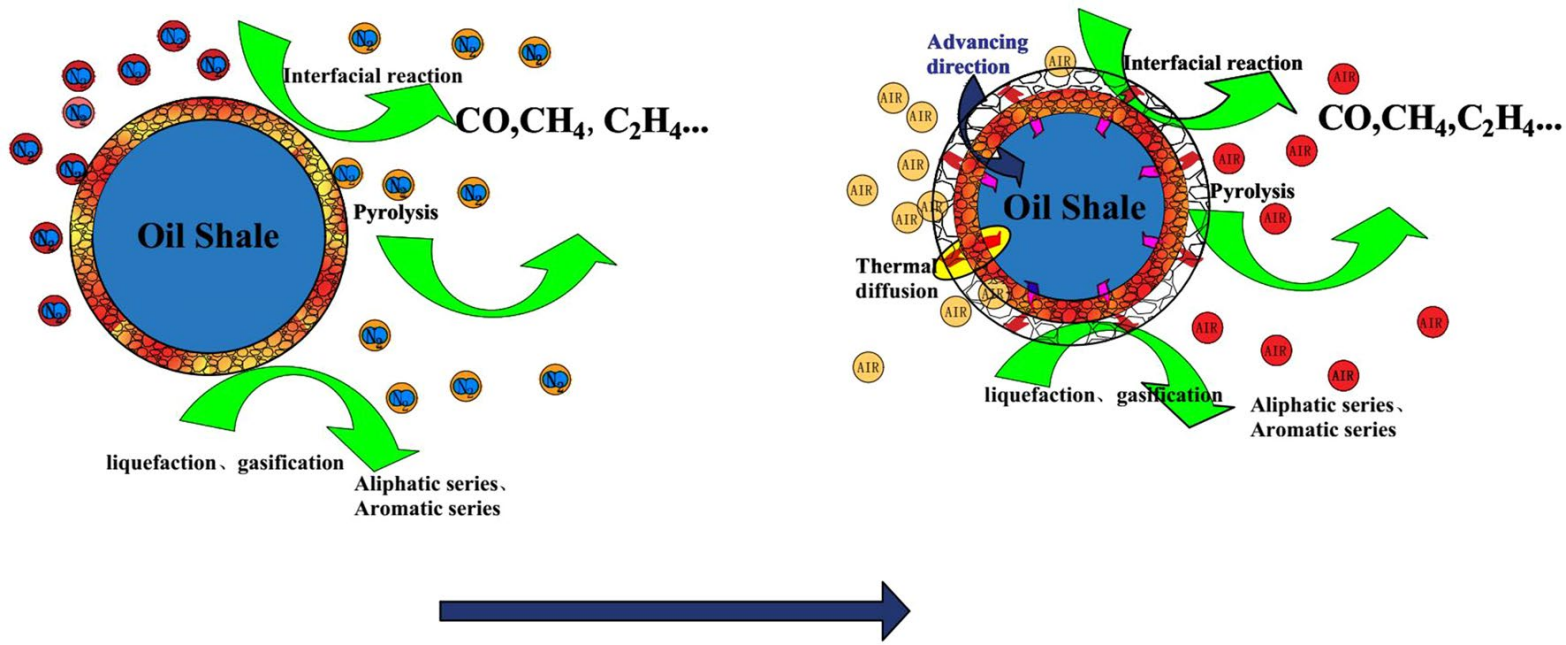
Schematic diagram of oil shale pyrolysis by topochemical heat.

In addition, compared with nitrogen atmosphere, the temperature range of kerogen pyrolysis in the second stage of oil shale pyrolysis is broadened in air atmosphere. According to the DTG curve, the second stage of oil shale pyrolysis in nitrogen atmosphere has a higher degree of aggregation, and the temperature change has a greater impact on the volatile release rate. However, in the air atmosphere, the release rate of volatile matter is relatively stable, and the maximum pyrolysis rate is also reduced to 0.1 compared with 0.21 of nitrogen, showing a uniform and slow release process throughout the second stage. This is because the oxygen in the air has a combustion supporting effect, which is a multiphase reaction synchronous propulsion process. As shown in Fig. 3, the organic matter and volatile matter in oil shale are burned in oxygen. The heat released by the reaction will lead to the increase of ambient temperature around oil shale particles, which will promote the temperature increase of surrounding particles, and promote the release of volatile matter and the combustion of organic matter. Therefore, the temperature range of the second stage has been widened in air, and the release rate of organic matter becomes slow and uniform.

**Figure 4:**
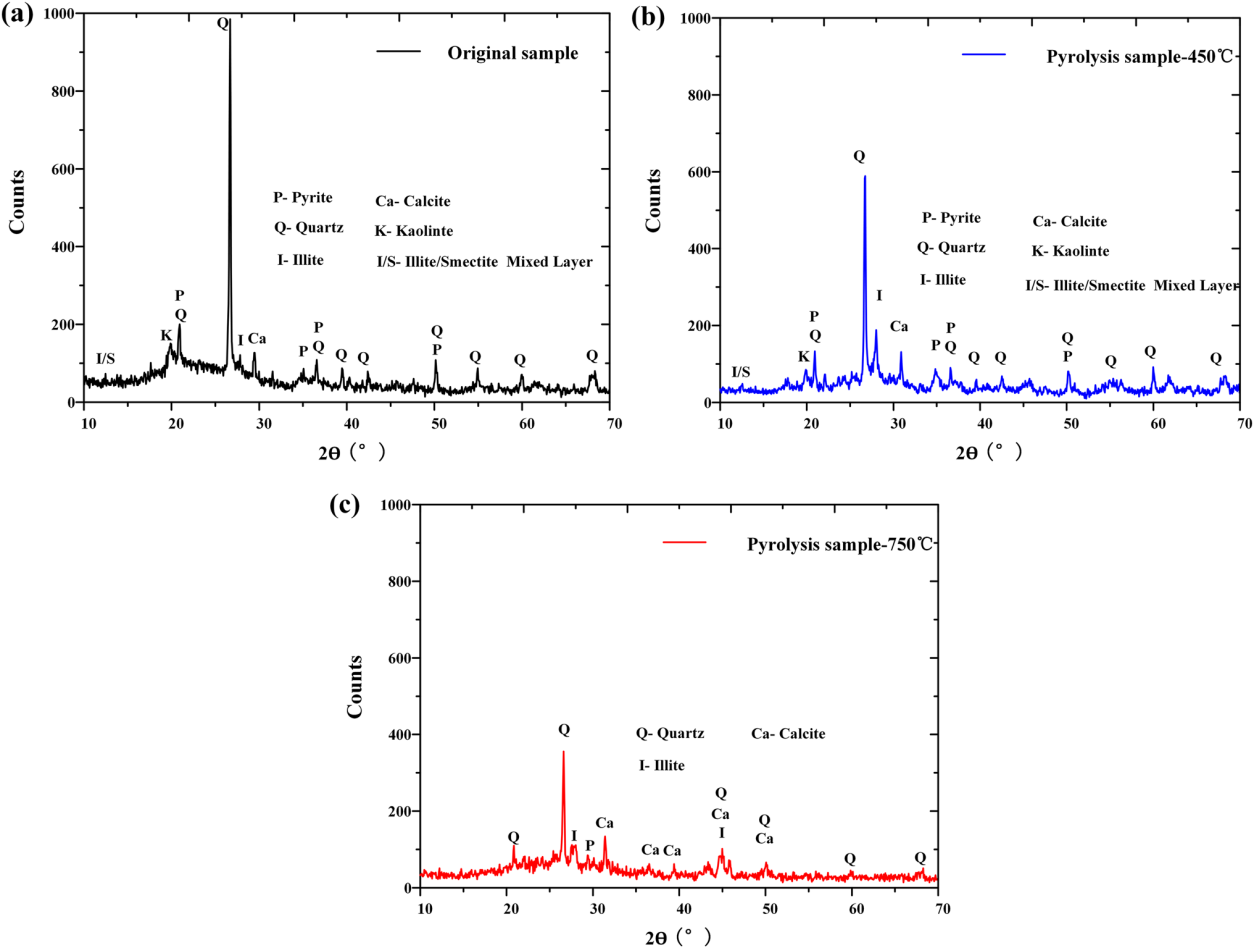
X-ray diffraction analysis of pyrolysis process of Fuyu oil shale.

As shown in Fig. 4, X-ray diffraction (XRD) analysis of pyrolysis process of Fuyu oil shale. When the temperature increases to 450 °C, the diffraction peaks of Kaolinite, Illite/Smectite mixed layer and pyrite decrease in varying degrees^26^. When the temperature rises to 750 °C, the above clay minerals decompose completely.

### Kinetic parameters calculations

The process of solving the kinetic parameters is to determine the activation energy and frequency factors that affect the reaction rate during the reaction process, and then reveal the control factors of the reaction, find the regularity of the reaction and realize the optimization of the actual process. The pyrolysis process of oil shale is a multiphase and multi-stage chemical reaction^21^. The simple model matching process does not accurately describe the complex heterogeneous pyrolysis system^22^. International Confederation for Thermal Analysis and Calorimetry (ICTAC) points out that the model free function method is more suitable for solving the dynamics of solid fuels^24^. In the kinetic analysis of the model free function method, KAS, FWO, Friedman is three most commonly used methods^23,24^. Therefore, the three methods were used to solve the pyrolysis kinetic parameters of Fuyu oil shale.

#### KAS method for solving activation energy E_a_

KAS method is the abbreviation of Kissinger–Akahira–Sunose, and its calculation method is as follows^9^.

1 $${\upalpha } = \frac{{m_{0} - m}}{{m_{0} - m_{\infty } }}$$

2 $$\ln \frac{\beta }{{T^{2} }} = \ln \left[ {\frac{{AE_{a} }}{G\left( \alpha \right)R}} \right] - \frac{{E_{a} }}{RT}$$

where, α-Conversion rate of oil shale pyrolysis in the second stage, %; m_0_—Initial mass of oil shale at the second pyrolysis stage, mg; m—sample mass at T K, mg; m_∞_—Final mass of oil shale at the second pyrolysis stage, mg; β—Heating rate, K·min^−1^; T—reaction temperature, K; R—gas constant, 8.314 J·mol^−1^; G(α)—Integral form of the most probable mechanism function; A—preexponential factor, s^−1^; E_a_—activation energy, kJ·mol^−1^.

It can be seen from Eq. (2) that $\ln \frac{\beta }{{T^{2} }}$ is a primary function of − $\frac{1}{T}$. By drawing $\ln \frac{\beta }{{T^{2} }}$ − (− $\frac{1}{T}$) at different conversion rates, the slope k can be obtained by fitting the curve with first order function. The activation energy E_a_ at the corresponding conversion rate can be obtained by k = E_a_/RT.

#### FWO method for solving activation energy E_b_

In order to further verify the accuracy of KAS method, the Flynn–Wall–Ozawa method was used to calculate the apparent activation energy E_b_ again^10^. The approximate formula of Doyle temperature integral was used, as shown in Eqs. (3) and (4).

3 $${\text{P}}\left( u \right) = 0.00484 \cdot e^{ - 1.051u}$$

4 $${\text{u}} = \frac{{E_{b} }}{RT}$$

The temperature approximation is introduced into the pyrolysis integral Eq. (5).

5 $${\text{G}}\left( \alpha \right) = \frac{{AE_{b} }}{\beta R} \cdot P\left( u \right)$$

The calculation formula of FWO method can be obtained, as shown in Eq. (6).

6 $${\text{lg}}\beta = {\text{lg}}\left( {\frac{{AE_{b} }}{RG\left( \alpha \right)}} \right) - 2.315 - 0.4567\frac{{E_{b} }}{RT}$$

As can be seen from Eq. (6), lgβ is a first-order function of − $\frac{1}{T}$. Through drawing lgβ − (− $\frac{1}{T}$) at different conversion rates, the slope k can be obtained by fitting the curve with the first-order function. The activation energy E_b_ can be calculated by k = 0.4576E_b_/RT.

#### Friedman method for solving activation energy E_c_

According to Arrhenius's law, the rate of reaction can be expressed as:

7 $$\frac{d\alpha }{{dt}} = \frac{A}{\beta }exp\left( { - \frac{E}{RT}} \right)$$

The logarithm of the two sides of the Eq. (1) is performed at the same time.

8 $$ln\frac{d\alpha }{{dt}} = {\text{ln}}\left( {A) - {\text{ln}}(\beta } \right) - \frac{E}{RT}$$

It can be seen from Eq. (2) that ln (dx/dt) − (− 1/T) curves are made for different conversion rates respectively. Slope k and intercept D. are obtained respectively, too. Activation energy E_c_ and pre-exponential factor A were calculated according to slope k and intercept D, respectively.

#### Inference of the most probable mechanism function from y(α)-α curve

The values of data α_i_, y(α)_i_(i = 0.05, 0.1…0.95) and a = 0.5, y(0.5) are brought into 41 groups of main functions of g(α) and corresponding f(α). The main function was calculated by y(α)^12^, and the curve of y(a)-a is made, which is regarded as the standard curve.

9 $${\text{y}}\left( {\upalpha } \right) = \frac{f\left( \alpha \right) \cdot G\left( \alpha \right)}{{f\left( {0.5} \right) \cdot G\left( {0.5} \right)}}$$

Then, the experimental data α_i_, T_i_, $\left( {\frac{d\alpha }{{dt}}} \right)_{i}$(i = 0.05, 0.1…0.95) and α = 0.5, T_0.5_,${ }\left( {\frac{d\alpha }{{dt}}} \right)_{0.5}$ were introduced into Eqs. (9) and (10) was obtained. The corresponding calculation of y(α) was also carried out, and the test curve of y(α)- α was drawn on the basis of the standard curve.

10 $${\text{y}}\left( {\upalpha } \right) = \left( {\frac{T}{{T_{0.5} }}} \right)^{2} \cdot \frac{{\left( {\frac{d\alpha }{{dt}}} \right)}}{{\left( {\frac{d\alpha }{{dt}}} \right)_{0.5} }}$$

If the test curve overlaps with the standard curve, or the data points of the test curve all fall on a certain standard curve. It can be determined that f(α) and g(α) corresponding to the curve are the most probable kinetic mechanism functions.

**Figure 5:**
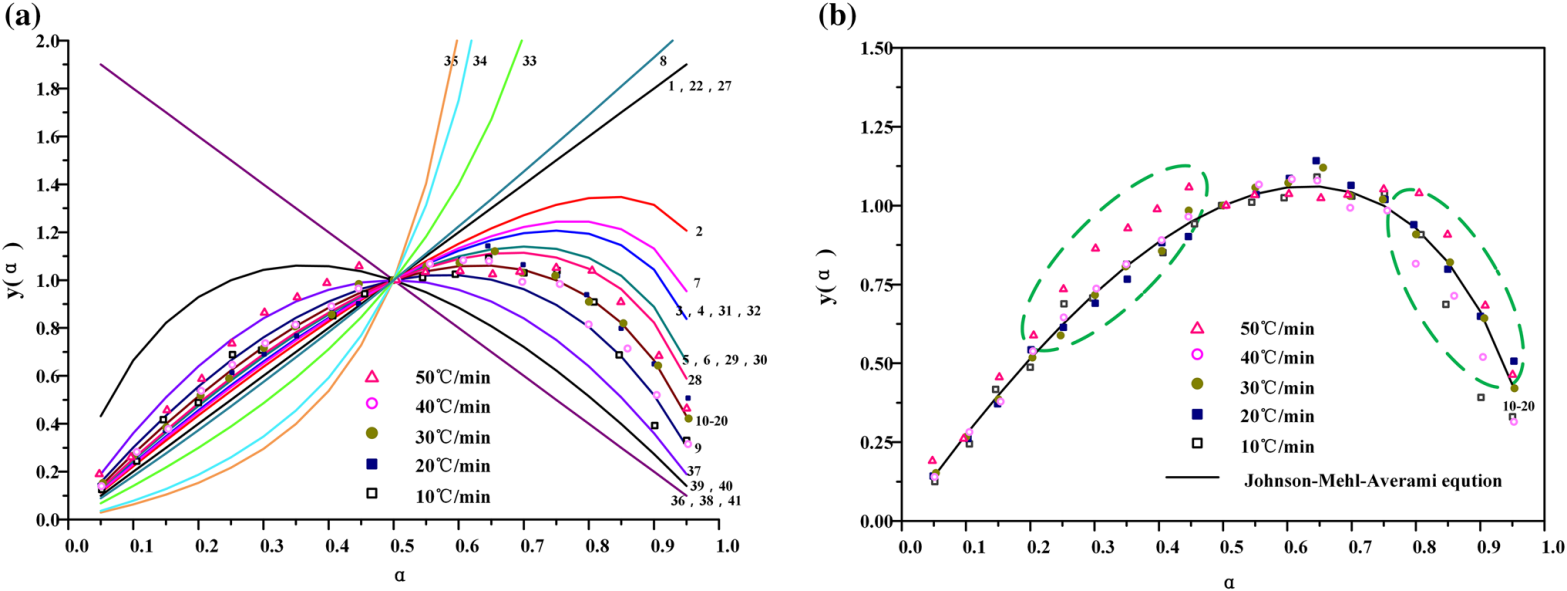
Standard curve and test curve of y(α)-α.

The standard curves and experimental curves of 41 mechanism functions y(α)- α are plotted by Malek method as shown in Fig. 5a. The experimental curves of Fuyu oil shale pyrolysis at different heating rates are distributed near Johnson–Mehl–Avrami equation. It shows that the pyrolysis mechanism function of Fuyu oil shale satisfies the reaction mechanism of random growth and subsequent nucleation. However, according to Fig. 5b, when the conversion is 20–40% and 70–90%, the dispersion between the test curve and the standard curve is greater. This is mainly reflected in the high heating rate corresponds to the low conversion interval, and the low heating rate corresponds to the high conversion interval. It may be that after random nucleation, small molecules of different chemical bond types are received at the growth stage. And because of the delayed heat transfer, the heat conduction products are released centrally, and secondary pyrolysis occurs after nucleation. Besides, for the Johnson–Mehl–Avrami reaction model, there are many different kinetic indices n. The pre-exponential factor A and other thermodynamic parameters can be calculated only if the kinetic exponent n is known exactly.

For Johnson–Mehl–Avrami reaction mode^14^,

11 $${\text{G}}\left( {\upalpha } \right) = \left[ {1 - ln\left( {1 - \alpha } \right)} \right]^{\frac{1}{n}}$$

12 $$f\left( {\upalpha } \right) = {\text{n}}\left( {1 - {\upalpha }} \right) \times [ - ln\left( {1 - \alpha } \right)]^{{1 - \frac{1}{n}}}$$

Combining with DTG curve, when the reaction rate reaches the maximum value,

13 $$- f^{\prime}\left( {\alpha_{P} } \right) \cdot {\text{G}}\left( {\alpha_{P} } \right) = 1$$

14 $$f^{\prime}\left( {\alpha_{P} } \right) = \left( {n - 1} \right)[ - ln\left( {1 - \alpha_{P} } \right)]^{{ - \frac{1}{n}}} - n\left[ { - ln\left( {1 - \alpha_{P} } \right)} \right]^{{1 - \frac{1}{n}}}$$

In the formula, $\alpha_{P}$ is the corresponding conversion at the peak of DTG curve, %.

Therefore, the kinetic exponent n can be obtained by combining the above three Eqs. (11)–(13).

15 $${\text{n}} = \frac{{1 - u_{P} \pi \left( {u_{P} } \right)}}{{ln\left( {1 - \alpha_{P} } \right) + 1}}$$

16 $$u_{P} = \frac{{E_{P} }}{{RT_{P} }}$$

In the formula, $E_{P}$ is the activation energy corresponding to the peak value of DTG curve, J·mol^−1^; $T_{P}$ is the temperature corresponding to the peak value of DTG curve, K;

According to Luke approximation^13^,

17 $${\uppi }\left( {u_{P} } \right) = \frac{{u_{P}^{3} + 18u_{P}^{2} + 86u_{P} + 96}}{{u_{P}^{4} + 20u_{P}^{3} + 120u_{P}^{2} + 240u_{P} + 120}}$$

In summary, the kinetic exponent n is calculated as shown in Table 2.

**Table 2:** Calculations of dynamic exponent n.

β/℃·min^−1^	10	20	30	40	50	Average
KAS	1.477	1.425	1.432	1.503	1.504	1.468
FWO	1.520	1.513	1.402	1.513	1.569	1.503
Friedman	1.629	1.681	1.687	1.521	1.718	1.647

### Solution of thermodynamic parameters

The activation energy E and the pre-exponential factor A obtained by kinetic calculation are substituted into Eq. (18) respectively. Arrhenius constant k^15^ at different heating rates can be obtained.

18 $${\text{lnk}} = {\text{lnA}} - \frac{E}{RT}$$

Then, the Arrhenius constant k was introduced into Eq. (19) to obtain Gibbs free energy ΔG^≠^ at different heating rates.

19 $$\Delta G^{ \ne } = RTln\frac{RT}{{Nhk}}$$

where T is the experimental temperature, K; R is the gas molar constant, 8.314 J·mol^−1^·K^−1^; N is the Avogadro constant, 6.024 × 10^23^ mol^−1^; h is the Planck constant, 6.625 × 10^−35^ J·s.

From the Eyring equation,

20 $${\text{ln}}\frac{k}{T} = \left( {\frac{{\Delta S^{ \ne } }}{R} + ln\frac{{k_{B} }}{h}} \right) - \frac{{\Delta H^{ \ne } }}{RT}$$

where k_B_ is a Boltzmann constant, 1.3807 × 10^–23^ ${\text{J}}\,\cdot\,K^{ - 1}$.

According to Eyring equation, the curve of ${\text{ln}}\frac{k}{T}$ − $\frac{1}{T}$ is plotted. The free enthalpy $\Delta H^{ \ne }$ can be obtained from the slope of the straight line. From the intercept of a straight line, free entropy $\Delta S^{ \ne }$ can be obtained.

## Results and discussion

### Kinetic process parameters of pyrolysis

According to the TG-DTG extrapolation method, the characteristic parameters of oil shale pyrolysis can be determined^16^. Specific steps are as follows: at the point of greatest gross loss rate on DTG curve of oil shale, make a vertical line intersecting with TG curve at point B. Make the tangent of the TG curve through point B. The tangent intersects the extension line of initial miss-focus A at a point C, and A is the initial miss-focus of the TG curve. The temperature corresponding to point C is the ignition point of oil shale. After passing the maximum weight loss rate of DTG curve, the horizontal line is made at a point D on the TG curve. Point D is a point at which the weight loss rate is reduced to a stable level after the second stage. The horizontal line intersects the tangent of point B with point E, which is the burnout temperature.

**Figure 6:**
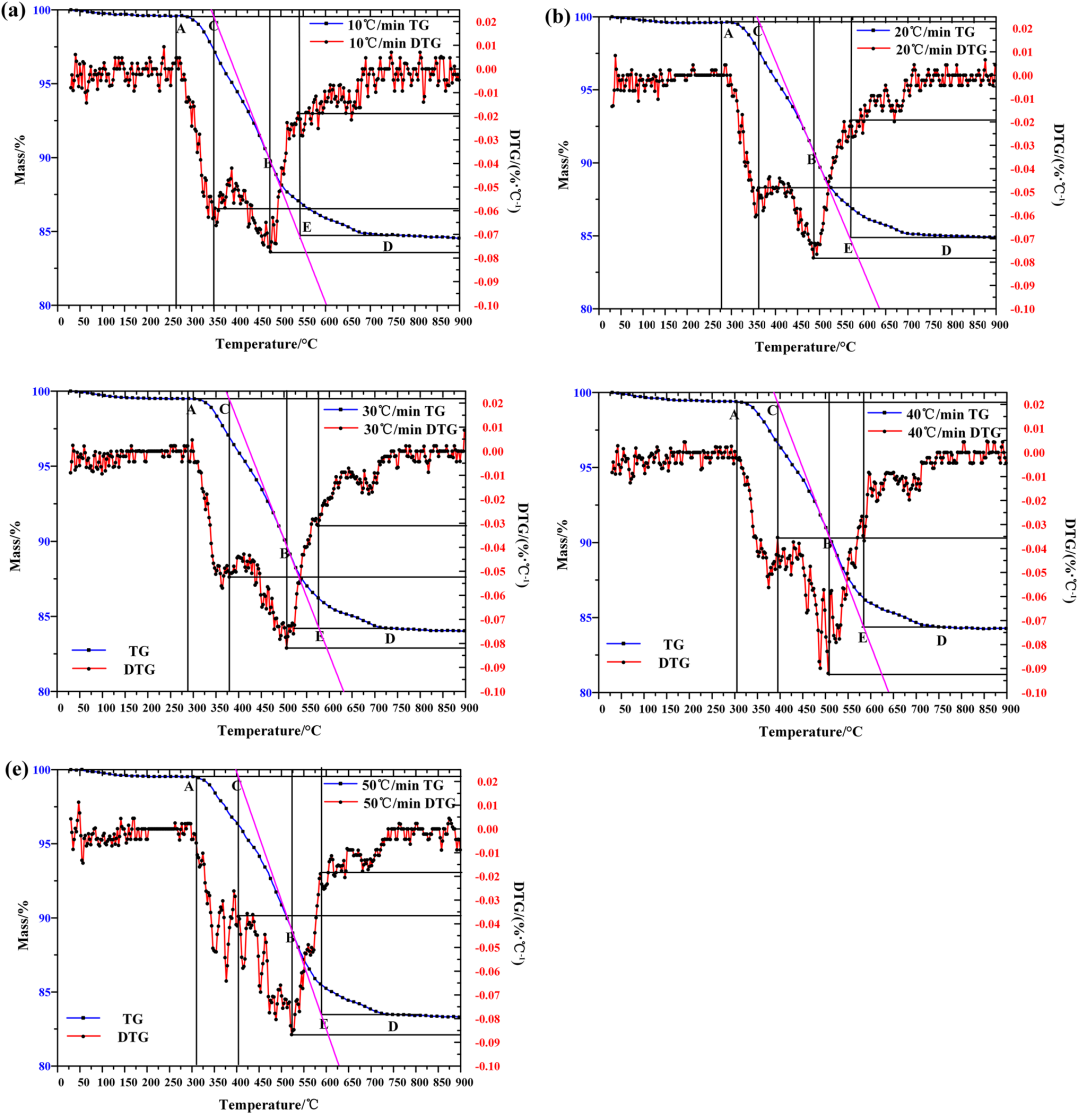
The process of extrapolation method for determining oil shale pyrolysis parameters.

As shown in Fig. 6, the extrapolation method is used to determine the pyrolysis parameters of oil shale in the second stage. The initial loss temperature T_O_, ignition temperature T_I_, maximum weight loss temperature T_P_, maximum weight loss rate (dm/dt) _max_ and burnout temperature Tc are obtained as shown in Table 3.

**Table 3:** Kinetic process parameters of Fuyu oil shale pyrolysis in air.

Area	β/(℃·min^−1^)	T_O_/℃	T_I_/℃	T_P_/℃	(dm/dt)_max_	Tc/℃
Fuyu	10	270	350	474	0.077	531
	20	277	360	486	0.079	549
	30	289	375	502	0.083	563
	40	300	392	507	0.093	577
	50	309	400	525	0.087	591

### Calculation of kinetic parameters

**Figure 7:**
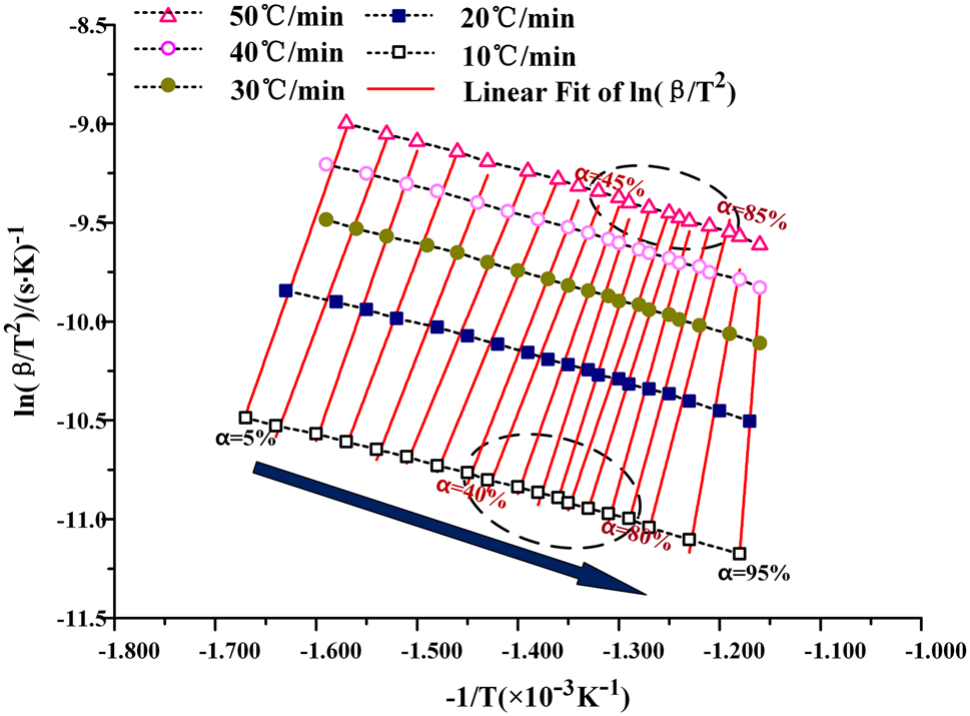
The KAS method for calculating activation energy.

**Figure 8:**
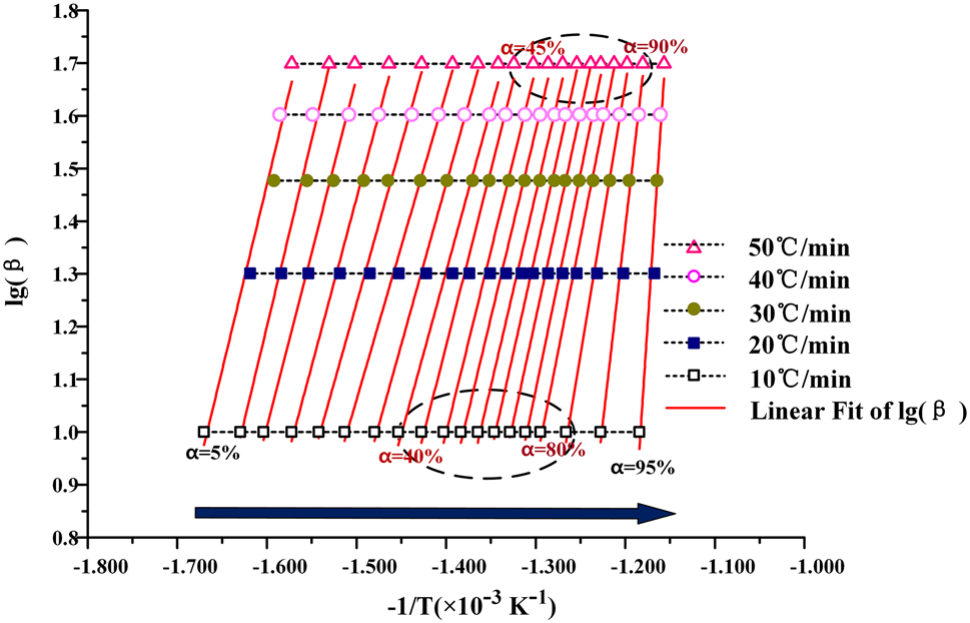
The FWO method for calculating activation energy.

**Figure 9:**
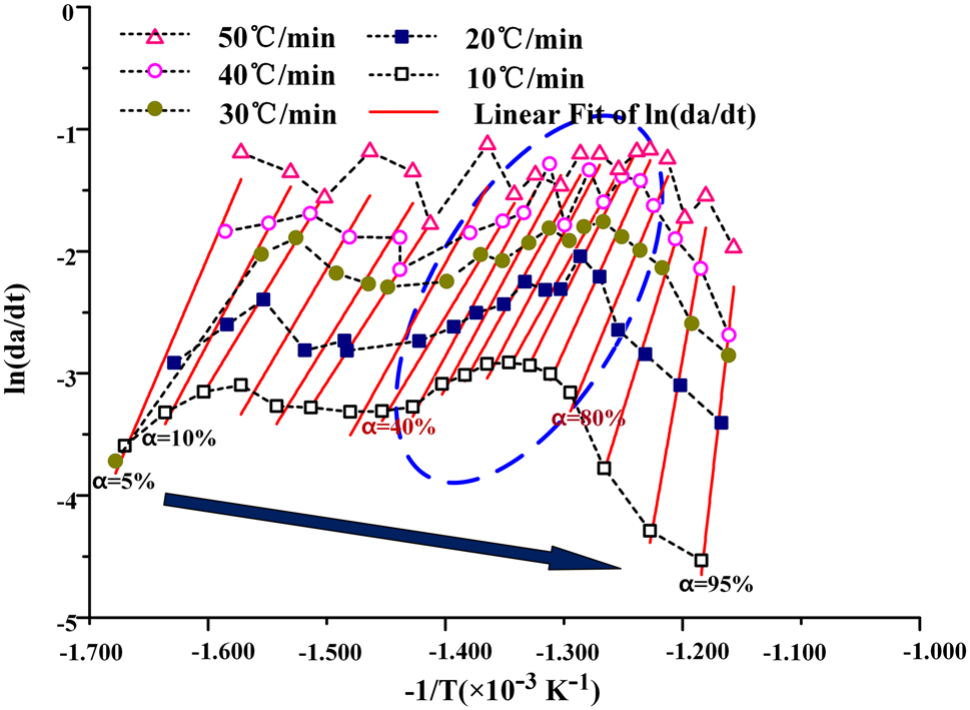
The Friedman method for calculating activation energy (**a**) nitrogen (**b**) air.

The KAS method, the FWO method and the Friedman method are used to calculate the kinetic curves of oil shale pyrolysis at the second stage, as shown in Figs. 7, 8 and 9 below. When the KAS method and the FWO method are used, the correlation coefficient r is greater than 0.98. But in the process of calculation by the Freidman method, the correlation coefficient is between 0.94 and 0.99, and the fitting degree of data is lower than that of KAS and FWO methods. In addition, the data fluctuate greatly during the fitting process, which indicates that Freidman method is not suitable for the calculation of pyrolysis kinetics of Fuyu oil shale.

When the heating rate is low, the area where oil shale pyrolysis is concentrated is between 40 and 80%, and the corresponding temperature is between 688 and 774 K. When the conversion rate is 80%, the maximum weight loss rate is reached. With the increase of heating rate, the area where oil shale pyrolysis is concentrated gradually moves to the area where conversion rate is 45–95%. This is due to the hysteresis of heat transfer. Oil shale belongs to heterogeneous system. In the process of pyrolysis, there is an active region of topo-reaction at the interface between the reactant and the product. The energy provided by the system for this area is larger than the activation energy of oil shale pyrolysis, so the oil shale in the active area pyrolysis first. With the continuous progress of pyrolysis, the oil shale in the topo-active region undergoes the random growth of products and the subsequent interfacial reaction of nucleation. The active region of the reaction interface is gradually advancing toward the direction of high conversion. According to the calculation results of the three methods, the activation energy of Fuyu oil shale pyrolysis at the second stage was obtained as shown in the Table 4 below.

**Table 4:** Activation energy of Fuyu oil Shale pyrolysis in air at the second stage.

α/%	KAS			FWO			Friedman		
	E/kJ·mol^−1^	A	R	E/kJ·mol^−1^	A	R	E/kJ·mol^−1^	A	R
10	116.529	1.95E+11	0.9816	129.166	2.44E+12	0.9885	152.570	1.06E+16	0.9766
20	110.992	1.87E+11	0.9903	115.788	2.43E+12	0.9939	136.923	1.03E+16	0.9465
30	100.217	1.88E+11	0.9932	106.176	2.26E+12	0.9944	101.447	1.03E+16	0.9550
40	106.328	1.99E+11	0.9829	112.422	2.43E+12	0.9859	132.874	1.14E+16	0.9746
50	119.979	1.93E+11	0.9894	125.775	2.52E+12	0.9911	149.153	0.97E+16	0.9420
60	126.872	1.92E+11	0.9898	132.649	2.41E+12	0.9914	152.961	1.05E+16	0.9547
70	136.790	1.89E+11	0.9977	142.390	2.36E+12	0.9980	161.349	1.11E+16	0.9973
80	151.381	1.93E+11	0.9941	154.735	2.47E+12	0.9949	194.797	1.10E+16	0.9467
90	266.821	1.95E+11	0.9838	269.281	2.43E+12	0.9836	456.414	1.06E+16	0.9717

**Figure 10:**
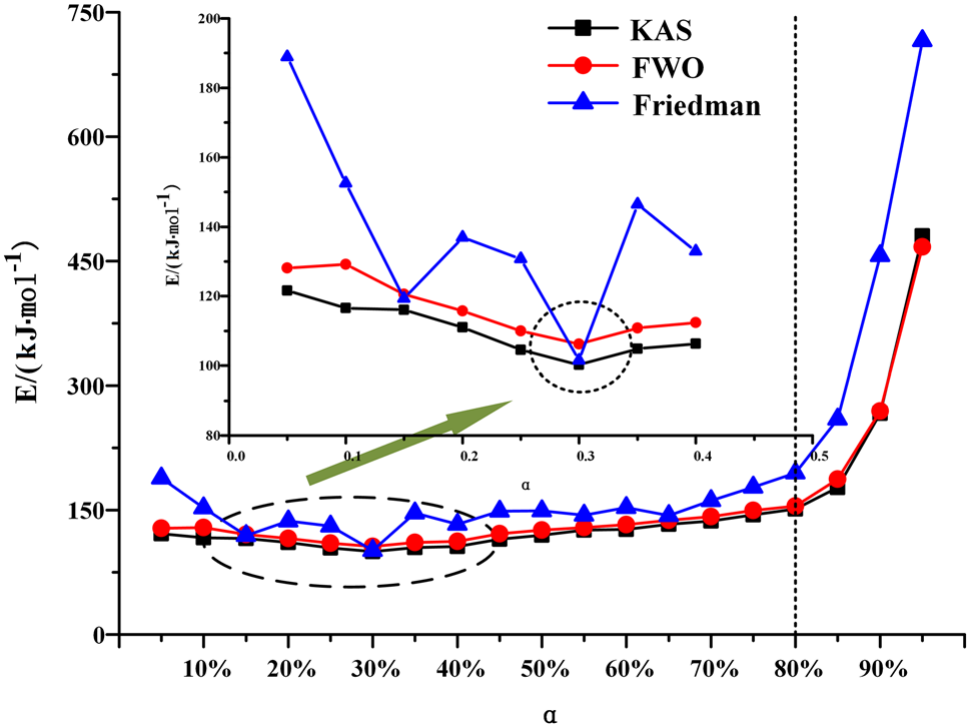
Curve of activation energy varies with conversion.

The second stage of oil shale pyrolysis is the main oil production stage. The activation energy of this stage is of great significance to the whole process and industrial production. The second stage conversion rate of oil shale pyrolysis is calculated based on Thermogravimetric curves. Since the activation energy is affected by the conversion rate, the corresponding activation energy is calculated every 5% of the conversion rate. The activation energy of oil shale pyrolysis at the second stage calculated by the three methods is shown in Fig. 10. With the increase of conversion, the activation energy decreases first and then increases. The overall activation energy increases with the increase of conversion rate during the second stage of oil shale pyrolysis. It is worth noting that when the conversion rate is 30%, all of them reach the minimum value. With the advancement of the second stage of oil shale pyrolysis, new macromolecule products will be pyrolysis and react with active molecule, resulting in the decrease of active molecule concentration. In addition, diffusion constraints and organic impurities also affect the activation energy. Bai et al. also have this phenomenon in the thermogravimetric analysis of different oxygen concentration in Huadian oil shale^25^. When the conversion rate is higher than 80%, the activation energy increases sharply. This is because the higher the conversion, the harder the reaction will occur. The activation energies obtained by KAS and FWO methods are relatively stable. The average activation energies of the second stage are 128.27 kJ·mol^−1^, 133.59 kJ·mol^−1^ respectively, when the conversion is 40–80%. However, the activation energy of oil shale pyrolysis at the second stage obtained by Friedman method reaches 158.23 kJ·mol^−1^, and fluctuates greatly with the increase of conversion rate.

### Thermodynamic decomposition characteristics

According to the transition state theory, kerogen needs to pass through a transition state in the pyrolysis process of oil shale, and then to produce oil and gas. In this transition process, it involves the complex coupling of multi-phase, multi-stage and multi-physical fields, and also includes the redistribution of energy and the rearrangement of chemical bonds. Thermodynamic parameters can explain the difficulty and spontaneity of this complex conditional reaction, especially the compensation effect of entropy enthalpy. The changes of free enthalpy ΔH^≠^, free entropy ΔS^≠^ and Gibbs free energy ΔG^≠^ during pyrolysis of oil shale can be obtained by calculation. It can be used to understand the degree of difficulty, spontaneity and reaction heat of oil shale pyrolysis. This provides a reference for in situ pyrolysis of oil shale by topo-chemical heat.

**Figure 11:**
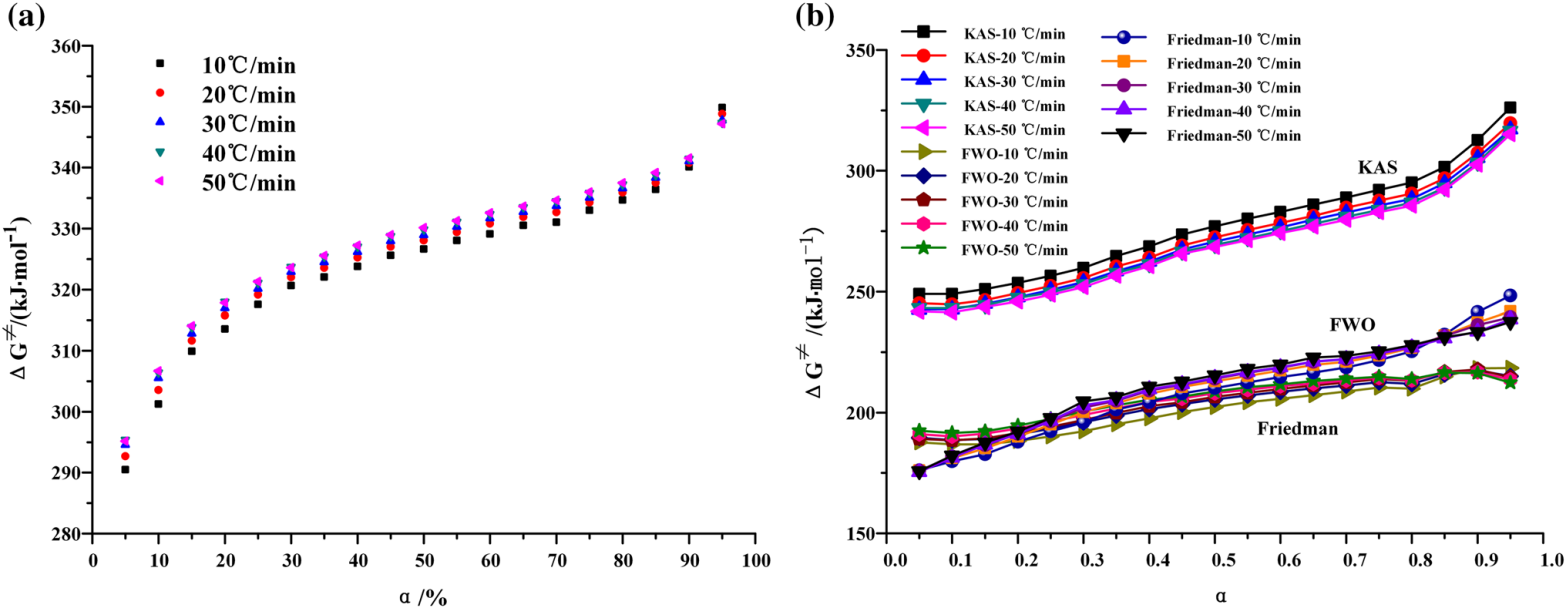
The Gibbs free energy ΔG^≠^ varies with conversion rate in stage II (**a**) nitrogen (**b**) air.

The Gibbs free energy ΔG^≠^ is the expression of the work done by the chemical reaction to the environment. As shown in Fig. 11, in the oil shale pyrolysis system, the work done by kerogen pyrolysis to the environment is the value of heat. That is to say, when the Gibbs free energy ΔG^≠^ of oil shale pyrolysis in nitrogen is greater than the activation energy E in air, the pyrolysis reaction of oil shale in air can be excited. At the same time, the Gibbs free energy ΔG^≠^ of oil shale pyrolysis in air is greater than the activation energy E, and the Topo-chemical method can advance spontaneously and continuously.

Transitional Gibbs free energy ΔG^≠^ is the most possible useful work to the environment during oil shale pyrolysis and the criterion of process spontaneity. The heating rate has little effect on Gibbs free energy. In these three different ways of calculation, with the increase of conversion and temperature, Gibbs free energy tends to increase. Besides, in the same calculation mode, when the conversion is the same, the increase of heating rate can cause the increase of Gibbs free energy, but the trend of this increase is weak, only in the KAS calculation process shows a slight obvious effect. The Gibbs free energy calculated by KAS method is the largest, with an average value of 271.82 kJ·mol^−1^. The Gibbs free energy calculated by FWO method is close to that calculated by Friedman method. The average Gibbs free energy calculated by FWO method is 204.12 kJ·mol^−1^, and the average Gibbs free energy calculated by Friedman method is 210.66 kJ·mol^−1^.

**Figure 12:**
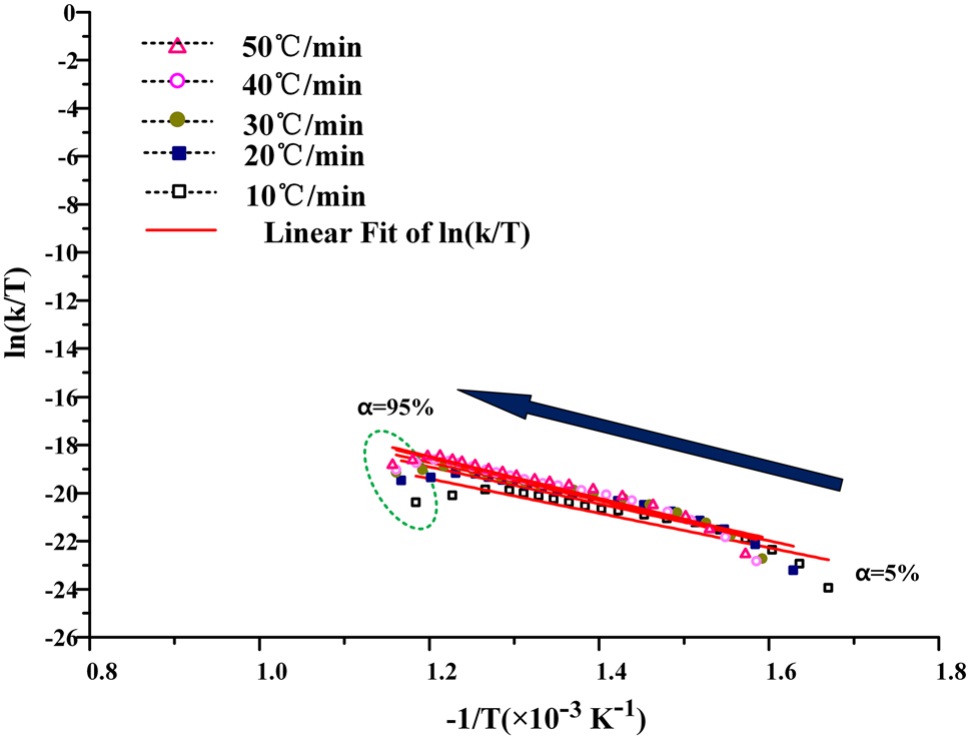
Process curves of free enthalpy and free entropy calculated by KAS method.

**Figure 13:**
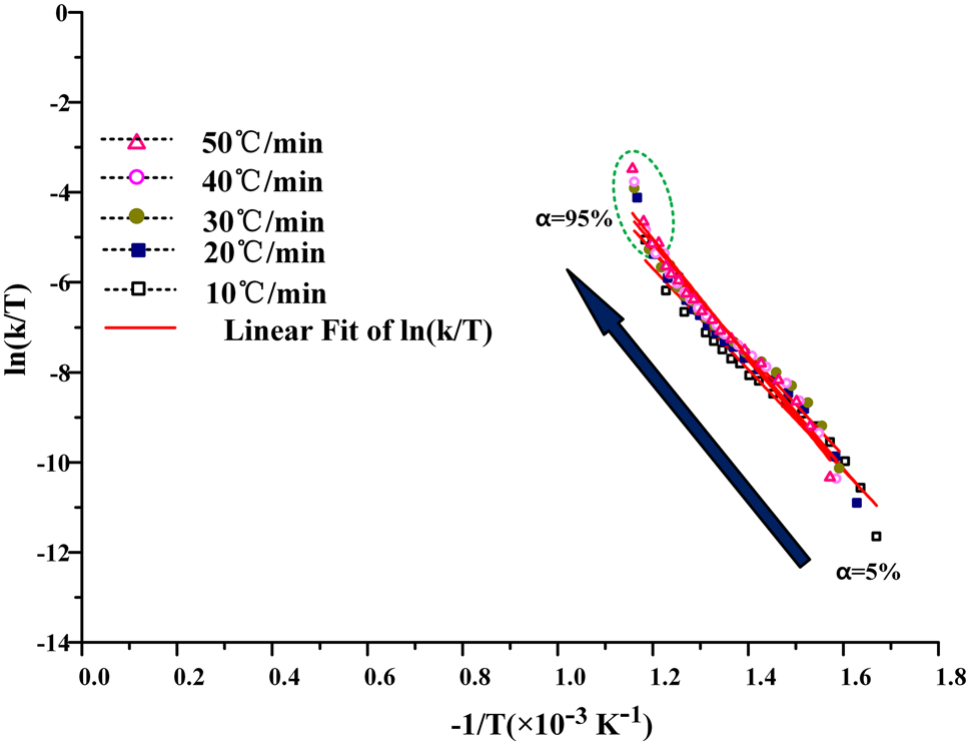
Process curves of free enthalpy and free entropy calculated by FWO method.

**Figure 14:**
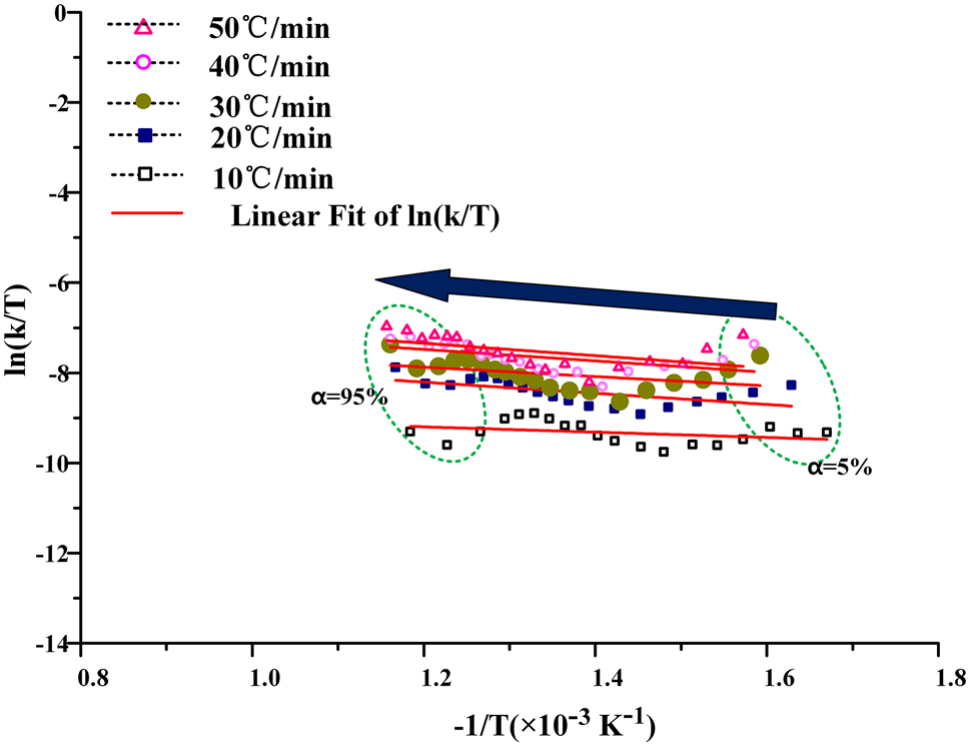
Process curves of free enthalpy and free entropy calculated by Friedman method.

In the process of calculating free enthalpy and free entropy by mapping ${\text{ln}}\frac{k}{T}$ − $\frac{1}{T}$, it is found that the data fitting curves of KAS and FWO methods are regular, as shown in Figs. 12 and 13. The experimental curves can be uniformly distributed near the fitting curve, and the fluctuation is small. But the ${\text{ ln}}\frac{k}{T}$ − $\frac{1}{T}$ curve obtained by Friedman method for calculating free enthalpy and free entropy fluctuates greatly, as shown in Fig. 14. The experimental curve is greatly affected by the heating rate, and the correlation between the experimental points and the fitting curve is also poor. This is because in the process of calculating activation energy by Friedman, the data cannot be well fitted. The subsequent calculation is based on the previous calculation, which is equivalent to magnifying the experimental error, so the data fitting degree is poor. When the free enthalpy and free entropy are calculated by KAS and FWO methods, only when the conversion rate is more than 95%, the obvious data dispersion phenomenon appears. This is because the extrapolation method is not accurate enough to calculate the pyrolysis interval of oil shale, which enlarges the temperature distribution interval of the second stage of oil shale pyrolysis. However, this does not affect the accuracy of data processing, because this paper focuses on the pyrolysis behavior of 40–80% conversion in industrial state.

The ${\text{ln}}\frac{k}{T}$ − $\frac{1}{T}$ curves of free enthalpy and free entropy calculated by KAS and FWO methods are shown in Figs. 12 and 13. The stripping fitting curve appeared at the test points with higher conversion. This is due to the uneven distribution of kerogen in the later stage of oil shale pyrolysis and the errors in judging the pyrolysis process and burnout point. It also shows that the calculation of burnout temperature by extrapolation method will enlarge the burnout temperature to some extent.

**Figure 15:**
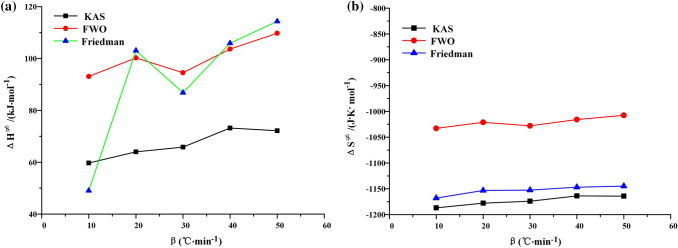
Free enthalpy and free entropy varies with heating rate.

The results of KAS, FWO and Friedman methods show that the free enthalpy and free entropy of oil shale pyrolysis at the second stage increase with the increase of heating rate, but this trend is weak. This indicates that the process can be carried out spontaneously at high temperature. As shown in Fig. 15a, the average free enthalpy calculated by KAS method is 66.98 kJ·mol^−1^, and the average free enthalpy obtained by FWO method is 100.25 kJ·mol^−1^. The Friedman method has a low degree of data fitting in the calculation process, and the calculation results fluctuate greatly, which has no value to reference.

As shown in Fig. 15b, the values of free entropy ΔS^≠^ calculated by KAS method and FWO method are negative. The average value of free entropy ΔS^≠^ calculated by KAS method is − 1173.31 J·mol^−1^·K^−1^, and the average value of free entropy ΔS^≠^ calculated by FWO method is − 1021.11 J·mol^−1^·K^−1^. This indicates that the orderliness of transition state increases and the degree of spontaneous reaction at low temperature is difficult, which is mainly related to the low oil content of Fuyu oil shale.

Combined with the above process, the continuous combustion of oil shale will release some internal energy to the environment. This measure of internal energy is free energy, which leads to high temperatures in the environment. High temperature conditions will react with the reaction system and transfer energy to the reaction system. When the energy transported is greater than the activation energy, the reaction can proceed spontaneously. In the pyrolysis process of Fuyu oil shale, the average activation free energy calculated by FWO method is 204.12 kJ·mol^−1^, which is much larger than 133.59 kJ·mol^−1^ calculated by FWO method. The average activation free energy calculated by KAS method is 271.82 kJ·mol^−1^, which is also much larger than the average activation energy calculated by KAS method in the second stage, 128.27 kJ·mol^−1^. Therefore, the reaction can continue spontaneously at high temperature.

### Volatile release characteristics

The product release characteristic index is a physical quantity describing the characteristics of volatile matter release during oil shale pyrolysis. Because the second stage of oil shale pyrolysis is the main stage of oil and gas generation, it is necessary to describe the effect of temperature and heating rate on oil and gas release during Topo-chemical heat pyrolysis oil shale, which provides an important reference for in-situ pyrolysis of oil shale.

In this paper, volatile release index I and reactive index R_a_ are used to describe the variation of pyrolysis product release characteristics with conversion and heating rate of oil shale in Fuyu area under non-isothermal conditions^17–19^. In addition, the related calculation is carried out at the conversion of 50% and 75% during the second stage of oil shale pyrolysis.

21 $${\text{I}}_{1/2} = \frac{{R_{a} }}{{T_{P} T_{i} \Delta T_{1/2} }}$$

22 $$\Delta T_{1/2} \to \frac{dm/dt}{{R_{a} }} = \frac{1}{2}$$

23 $${\text{I}}_{3/4} = \frac{{R_{a} }}{{T_{P} T_{i} \Delta T_{3/4} }}$$

24 $$\Delta T_{3/4} \to \frac{dm/dt}{{R_{a} }} = \frac{3}{4}$$

25 $$R_{a} = - \frac{1}{{m - m_{\infty } }}\frac{dm}{{dt}} = \frac{1}{1 - a}\frac{da}{{dt}}$$

∆T_1/2_—the temperature range corresponding to 50% conversion, K; also called peak width. ∆T_3/4_—the temperature range corresponding to the conversion of 75%, K.

The above equation reflects the characteristics of volatile matter released by instantaneous organic matter transformation in the process of oil shale pyrolysis to 50% and 75%.

**Figure 16:**
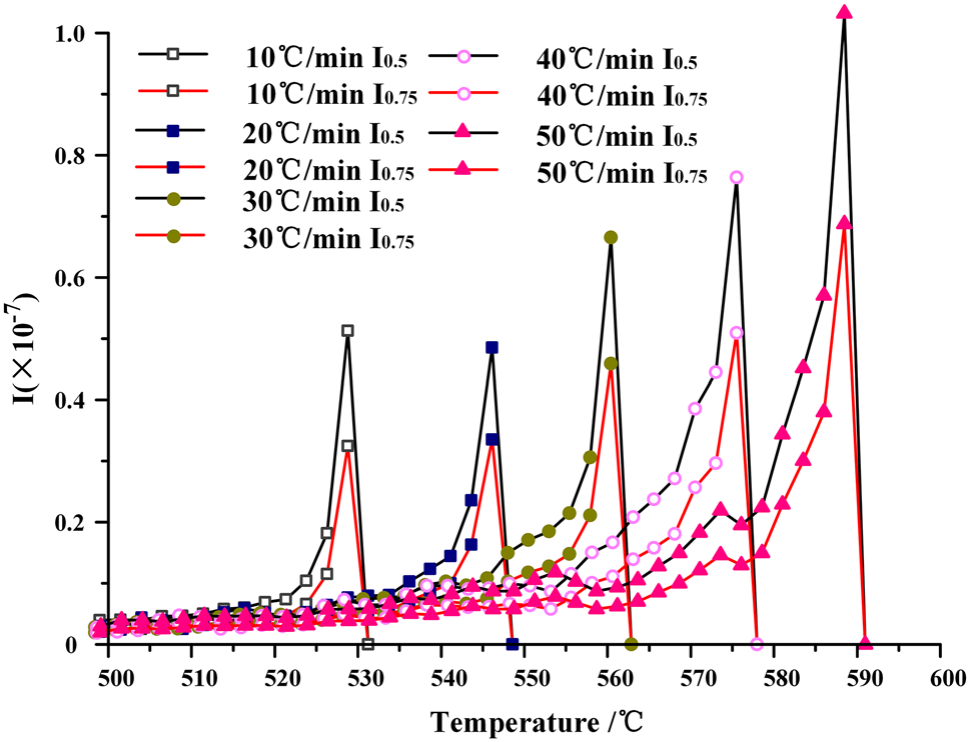
Volatile release characteristic index curve.

From Fig. 16, it can be seen that the volatile release index in the second stage is significantly affected by the heating rate. At the same heating rate, there is no difference between the pyrolysis interval corresponding to 50% and 75%, but the product release characteristic index corresponding to 50% conversion is more than 75%. This also indicates that the higher the conversion, the more difficult the reaction will occur in the same pyrolysis temperature interval. With the increase of heating rate, pyrolysis moves to high temperature zone, and the volatile release index at the same conversion rate increases, too. When the heating rate is 10 °C/min, the volatile release index corresponding to 75% conversion is 0.3 × 10^–7^, and when the heating rate reaches 50 °C/min, it reaches 0.7 × 10^–7^. This is because the pyrolysis process of kerogen is a process of chemical bond breaking and recombination. With the increase of heating rate, the rate of interfacial reaction advancing into the interior of oil shale particles will increase, which will lead to more complex reaction process. The results show that the oil and gas products released by kerogen pyrolysis in the outer layer of oil shale particles have not yet been displaced, the temperature gradient increases, and the interfacial reaction has been pushed to the interior of oil shale particles, resulting in more kerogen pyrolysis. The multi-stage parallel reaction is carried out simultaneously, so the release index of volatile matter will also increase. While partially removed side chains and small molecular structures cannot be further decomposed, the temperature of pyrolysis system has risen, and more macromolecular substances have been precipitated. Therefore, this part of the material has to be pyrolyzed together with other macromolecule materials in the high temperature region, so the region where the reaction is concentrated gradually migrates to the high temperature region.

## Conclusion

1. Based on the transition state theory, KAS method and FWO method are used to calculate the changes of free enthalpy, free entropy and Gibbs free energy in the pyrolysis process of oil shale. The average Gibbs free energy ΔG^≠^ obtained by FWO method is 204.12 kJ·mol^−1^, the average activation enthalpy ΔH^≠^ is 100.25 kJ·mol^−1^, and the average activation entropy ΔS^≠^ is − 1021.11 J·mol^−1^·K^−1^.
2. At high temperature, when the Gibbs free energy ΔG^≠^ of oil shale pyrolysis in nitrogen is greater than the activation energy E in air, the pyrolysis reaction of oil shale in air can be excited. At the same time, the Gibbs free energy ΔG^≠^ of oil shale pyrolysis in air is greater than the activation energy E, and the Topo-chemical method can advance spontaneously and continuously.
3. With the increase of heating rate, the oil shale pyrolysis progress moves to the high temperature zone, and at the same conversion rate, the volatile emission index also showed an increasing trend.
